# Synthesis and structure of an aryl­selenenium(II) cation, [C_34_H_41_N_4_Se^+^]_2_[Hg(SeCN)_4_]^2−^, based on a 5-*tert*-butyl-1,3-bis­(1-pentyl-1*H*-benzimidazol-2-yl)benzene scaffold

**DOI:** 10.1107/S2056989018006394

**Published:** 2018-05-15

**Authors:** Varsha Rani, Harkesh B. Singh, Ray J. Butcher

**Affiliations:** a Department of Chemistry, Indian Institute of Technology Bombay, Powai, Mumbai 400 076, India; b Department of Chemistry, Howard University, 525 College Street NW, Washington, DC 20059, USA

**Keywords:** crystal structure, aryl­selenenium(II) cation, tetra­seleno­cyanate­mercury(II) anion

## Abstract

In the title salt, bis­{[5-*tert*-butyl-1,3-bis­(1-pentyl-1*H*-benzimidazol-2-yl)benzene]selenium} tetra­kis­(seleno­cyanato)­mercury, (C_34_H_41_N_4_Se)_2_[Hg(SeCN)_4_], the aryl­selenenium cations, [C_34_H_41_N_4_Se]^+^, are linked through [Hg(SeCN)_4_]^2−^ anions by C—H⋯N hydrogen bonds. In the cation, the geometry around the Se atom in the 5-*tert*-butyl-1,3-bis­(1-pentyl-1*H*-benzimidazol-2-yl)benzene scaffold is T-shaped, resulting from the coordination of Se by the C atom of the central aromatic ring and the N atoms of both of the benzimidazole moieties. The *trans* Se—N bond lengths are almost equal [2.087 (3) and 2.099 (3) Å] and the Se—C bond length is 1.886 (3) Å. The N—Se—N angle is 159.29 (11)°. The geometry around the Hg^II^ atom in the [Hg(SeCN)_4_]^2−^ anion is distorted tetra­hedral, with Se—Hg—Se angles ranging from 88.78 (3) to 126.64 (2)°. In [Hg(SeCN)_4_]^2−^, the Hg—Se bonds are unsymmetrical [2.5972 (4) and 2.7242 (5) Å]. One of the pentyl substituents is disordered over two equivalent conformations, with occupancies of 0.852 (8) and 0.148 (8).

## Abstract 2

In [C_34_H_41_N_4_Se^+^]_2_[Hg(SeCN)_4_]^2−^, the aryl­selenenium cations, [C_34_H_41_N_4_Se]^+^, and [Hg(SeCN)_4_]^2−^ anions are linked by C—H⋯N hydrogen bonds. In the cation, the geometry around the Se atom is T-shaped, resulting from the coordination of Se by the C atom of the central aromatic ring and the N atoms of the benzimidazolyl moieties.

## Chemical context   

Over the past two decades, organochalcogen chemistry has gained the attention of synthetic chemists because of its promising utility in biomimetic chemistry (Mugesh & Singh, 2000 ▸; Zhao *et al.*, 2012 ▸; Bhuyan & Mugesh, 2012 ▸), synthetic organic chemistry (Back 1999 ▸; Singh & Wirth, 2012 ▸; Chivers & Laitinen, 2015 ▸) and material science (Manjare *et al.*, 2014 ▸; Kremer *et al.*, 2015 ▸). The first stable selenenium cation complex, [2,6-(Me_2_NCH_2_)_2_C_6_H_3_Se]^+^[PF_6_]^−^, was isolated while attempting the synthesis of the respective oxides from the reaction of 2,6-bis­[(di­methyl­amino)­meth­yl]phenyl methyl selenide with *t*-BuOCl (Fujihara *et al.*, 1995 ▸). In the literature, examples of aryl­selenenium(II) cations are limited to a basic scaffold, the [2,6-bis­(di­methyl­amino­meth­yl)phen­yl]sel­enen­ium moiety, which is stabilized by different counter-anions [Cl^−^, Br^−^, I^−^ (Pop *et al.*, 2014 ▸) and HF_2_^−^ (Poleschner & Seppelt, 2004 ▸)].

Our group has been active in the area of synthesis and isolation of novel, unstable aryl­chalcogen derivatives featuring intra­molecular inter­actions (*E*⋯*D*; *E* = S, Se, Te and *D* = N, O) between chalcogen heteroatoms by using either one or two coordinating groups (Zade *et al.*, 2004*a* ▸,*b* ▸; Selvakumar *et al.*, 2011*a* ▸,*b* ▸,*c* ▸,*d* ▸; Singh *et al.*, 2011 ▸; Prasad *et al.*, 2016 ▸). Recently, and for the first time, we have shown the use of the bis-benzimidazole group to isolate an organometallic derivative of a non-transition metal where 1,3-bis­(N-substituted benzimidazol-2′-yl)benzene has been used as a pincer ligand with chalcogens (Rani *et al.*, 2018*a* ▸).

As far as the synthesis of transition metal complexes with the bis-benzimidazole group is concerned, there are several reports in the literature for platinum(II) pincer complexes with similar kinds of scaffolds. Some of these were investigated for their photoluminescence properties (Wang *et al.*, 2014 ▸; Dorazco-González, 2014 ▸; Chan *et al.*, 2016 ▸). Recently, we also reported some palladium(II) pincer complexes with a 1,3-bis­(N-substituted benzimidazol-2′-yl)benzene-based ligand. In all the cases, we found that the transition metal complexes were quite stable and in no case was auto-ionization observed (Rani *et al.*, 2018*b* ▸).

In an attempt to synthesize {4-(*tert*-but­yl)-2,6-bis­(1-pentyl-1*H*-benzo[*d*]imidazol-2-yl)phen­yl}(seleno­cyanato)­mercury (**3**), [4-*tert*-butyl-2,6-bis­(1-pentyl-1*H*-benzimidazol-2-yl)phen­yl]mercury(II) chloride (**1**) was reacted with potassium seleno­cyanate in 1,4-dioxane under reflux conditions. It was observed that, instead of the formation of the desired compound, the reaction leads to the isolation of an aryl­selenenium(II) cation *via* auto-ionization (Scheme 1). The procedure for the synthesis of complex **1** will be reported elsewhere. A plausible mechanism for the formation of complex **2** is shown in Scheme 2. Organomercury complex **1** reacts with potassium seleno­cyanate to form the desired product **3** with potassium chloride as a by-product. However, if complex **II** is unstable, mercury may be eliminated in elemental form *via* a reductive elimination pathway to form inter­mediate **III**. Strong secondary bonding inter­actions between Se⋯N atoms may facilitate auto-ionization and the formation of an aryl­selenenium cation with CN^−^ as the counter-anion **IV**. In the presence of a polar protic solvent, there is the possibility of decomposition of organomercury complex **1** to give the free ligand along with HgCl_2_ and Hg(OMe)_2_ as by-products.
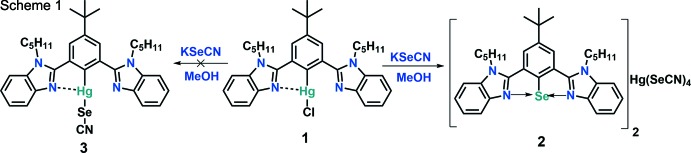


HgCl_2_ reacts with an excess of KSeCN to form K_2_[Hg(SeCN)_4_] (Space & Armeanu, 1930 ▸). Two selenenium cations can then associate with the [Hg(SeCN)_4_]^2−^ anion to form complex **2**. Since we only used one equivalent of potassium seleno­cyanate for the reaction, the product was obtained in low yield (11%).
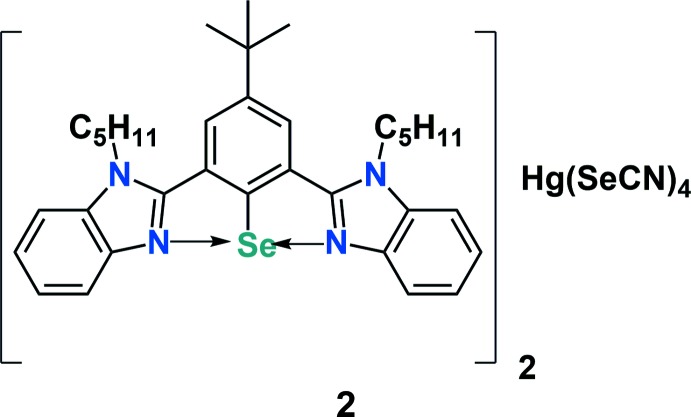
 
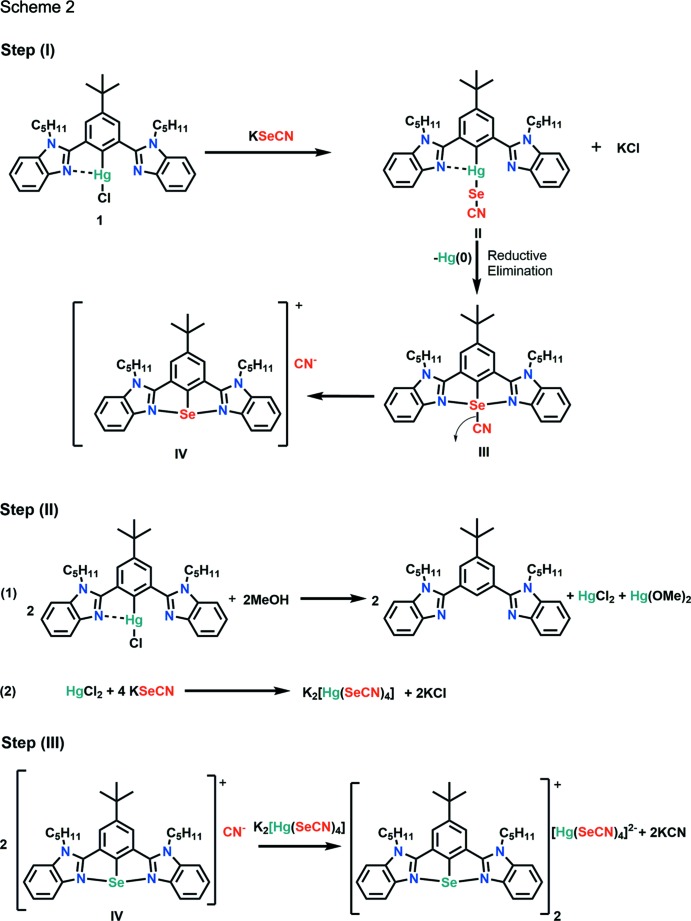


## Structural commentary   

**Figure 1:**
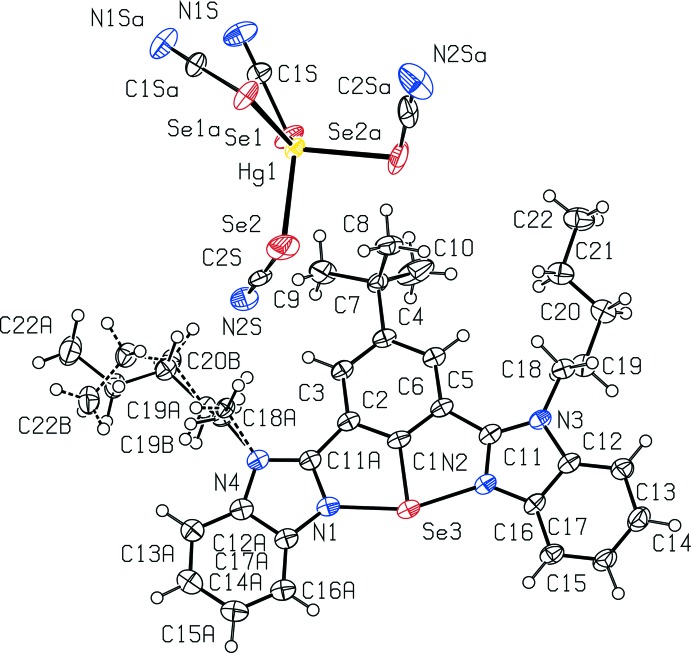
A view of the structure of the title compound, showing the atom-labelling scheme and the disorder in the pentyl side chain. Displacement ellipsoids are drawn at the 50% probability level. Symmetry code for generating equivalent atoms: 1 − *x*, *y*, 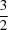 − *z*.

The title compound, **2**, crystallizes in the monoclinic space group *C*2/*c*. The asymmetric unit contains a selenenium cation along with half of a [Hg(SeCN)_4_]^2−^ anion with the Hg atom located on a crystallographic twofold axis (Fig. 1 ▸). In the cation, the coordination geometry around Se is T-shaped with each Se atom bonded to the central carbon atom of the aromatic ring and intra­molecularly coordinated to the two N atoms. This coordination gives rise to a hepta­cyclic framework. The tetra­cyano­seleno­mercurate anion [Hg(SeCN)_4_]^2−^ is sandwiched between two aryl­selenenium cationic units. The observed Se—C bond length is 1.886 (3) Å, which is comparable with that found for a NCN pincer-based selenenium cation [2,6-(Me_2_NCH_2_)_2_C_6_H_3_Se]^+^[PF_6_]^−^ (1.874 Å; Furukawa *et al.*, 1995 ▸), and an OCN pincer-based selenenium cation [2-NO_2_,6-(C_6_H_5_N=CH)C_6_H_3_Se]^+^[Br_3_]^−^ (1.84 Å). The Se3—N1 and Se3—N2 bond lengths are almost equal [2.087 (3) and 2.099 (3) Å]. The Se—N distances are shorter than the sum of the van der Waals radii for Se and N [Σ_rvdw_(Se,N) 3.45 Å] and longer than the covalent radii [Σ_rcov_(Se,N) 1.91 Å] (Bondi, 1964 ▸). This implies stronger intra­molecular Se⋯N inter­actions in the selenenium cation. The N1—Se3—N2 bond angle is found to be 159.29 (11)°. In related mol­ecules (Rani *et al.*, 2017*a* ▸,*b* ▸,*c* ▸), in the absence of coordinated Hg or Se atoms, the benzimidazole arms are twisted significantly out of the plane of the central phenyl ring. However, in the present structure, as a result of the inter­action with Se, the two benzimid­azole arms are almost in the plane of the central phenyl ring [dihedral angles of 3.10 (16) and 7.18 (19)°]. The Se atom is displaced by 0.116 (4) Å from the plane of the central phenyl ring. The atoms involved in the chelating system (N2, C11, C6, C1, C2, C11*A*, N1) form a plane (r.m.s deviation for fitted atoms of 0.0182 Å) with the Se in this plane [deviation from the plane of 0.011 (2) Å].

In the anion, the mercury atom is coordinated by four seleno­cyanate anions (two are crystallographically unique) and the geometry around the mercury atom is distorted tetra­hedral with Se—Hg—Se angles ranging from 88.78 (3) to 126.64 (2)°. The tetra­cyano­seleno­mercurate anion [Hg(SeCN)_4_]^2−^ acts as a bridging moiety between two selenenium cationic units. The Se3⋯Se2(−*x*, −*y*, 1 − *z*) distance is 4.189 (2) Å and the C1—Se3⋯Se2(−*x*, −*y*, 1 − *z*) angle is 163.40 (9)°, which indicates that there is a weak secondary inter­action between the two different kinds of Se atoms in the cation and anion (Se3 and Se2). In the [Hg(SeCN)_4_]^2−^ anion, two sets of Hg—Se bonds exist. One set is shorter [2.5972 (4) Å] and the other set is longer [2.7242 (5) Å]. The Hg–SeCN moieties are not linear, with Hg—Se—C angles of 101.31 (14) and 101.43 (11)°.

## Supra­molecular features   

**Figure 2:**
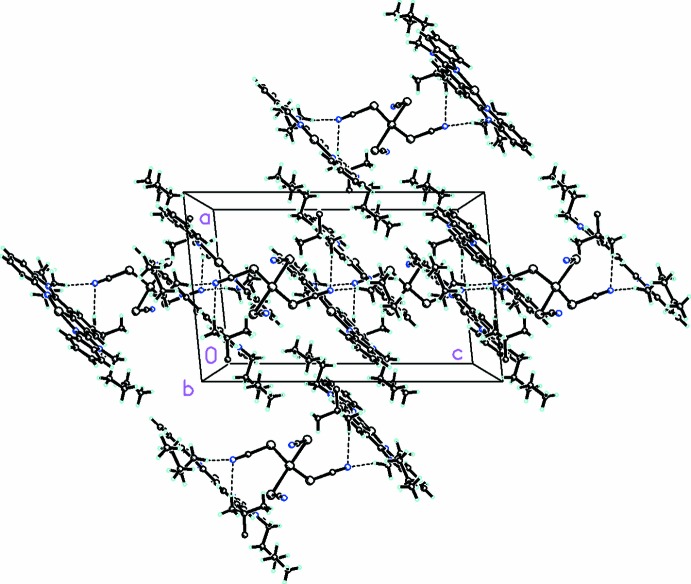
Packing diagram viewed along the *b* axis. C—H⋯N inter­actions linking the cations and anions are shown as dashed lines. Only the major disorder component is shown for clarity.

In the crystal, the mol­ecules are arranged in a parallel fashion along the *b*-axis direction as shown in Fig. 2 ▸. These parallel units are stacked together by C18—H18*A*⋯N1*S* and C18*A*—H18*C*⋯N2*S* inter­actions (numerical details are given in Table 1 ▸) and π–π stacking inter­actions between the benzimidazole rings (centroid–centroid distances = 3.535 Å).

## Database survey   

There are no structural reports in the literature on a [phenyl­enebis(benzimidazole)]selenenium cation. However, there have been several reports of structures containing [Hg(SeCN)_*x*_]^2−^ moieties [CICLOP, Brodersen *et al.* 1984 ▸; LENHES, Li *et al.*, 2006*a* ▸; LENHES01, Sun *et al.*, 2005 ▸; MURQOH, Li *et al.*, 2006*b* ▸; PUMVAU, Kushch, *et al.*, 1998 ▸; WUYGUU, Sun *et al.*, 2013 ▸; YIHKUV, Shibaeva *et al.* 1994 ▸; YIHKUV01, Shibaeva *et al.* 1997 ▸]

## Synthesis and crystallization   

To a solution of **1** (0.2 g, 0.269 mmol) in 1,4-dioxane (30 ml) was added potassium seleno­cyanate (0.039 g, 0.270 mmol) dissolved in MeOH. The reaction mixture was stirred for 6 h under a nitro­gen atmosphere and refluxed. The reaction mixture was filtered and the precipitate was washed with dioxane. Colourless prism-shaped crystals of **2** were obtained by layering a MeOH solution with diethyl ether at room temperature.

Yield 11% (0.058 g, 0.036 mmol); m. p. turned blackish after 423 K was reached. FT–IR (KBr) (cm^−1^): 3059 (*w*), 2957 (*s*), 2931 (*s*), 2869 (*s*), 2124 (*s*, needle-like, C≡N), 1614 (*m*), 1464 (*s*), 1458 (*s*), 1440 (*s*), 1330 (*w*), 1288 (*w*), 1273 (*w*), 1154 (*w*), 1137 (*w*), 1011 (*w*), 892 (*w*), 746 (s). ESI–MS: *m*/*z* calculated for C_34_H_41_N_4_Se: 585.2496. Found: 585.2552.

## Refinement   

Crystal data, data collection and structure refinement details are summarized in Table 2 ▸. The H atoms were positioned geometrically and allowed to ride on their parent atoms, with C—H distances ranging from 0.95 to 0.99 Å. *U*_iso_(H) = xU_eq_(C), where *x* = 1.5 for methyl H atoms and 1.2 for all other C-bound H atoms. One of the pentyl substituents is disordered with an occupancy ratio of 0.852 (8):0.148 (8). It was refined as two equivalent conformations using SAME and SIMU instructions (SAME 0.01 and SIMU 0.01).

## Supplementary Material

Crystal structure: contains datablock(s) I. DOI: 10.1107/S2056989018006394/zl2726sup1.cif

Structure factors: contains datablock(s) I. DOI: 10.1107/S2056989018006394/zl2726Isup2.hkl

CCDC reference: 1839609

Additional supporting information:  crystallographic information; 3D view; checkCIF report

## Figures and Tables

**Table 1:** Hydrogen-bond geometry (Å, °)

*D*—H⋯*A*	*D*—H	H⋯*A*	*D*⋯*A*	*D*—H⋯*A*
C18—H18*A*⋯N1*S* ^i^	0.99	2.62	3.568 (5)	160
C18*A*—H18*C*⋯N2*S*	0.99	2.38	3.324 (8)	159
C18*B*—H18*F*⋯N2*S*	0.99	2.22	3.06 (6)	142

Symmetry code: (i) 
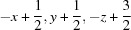
.

**Table 2:** Experimental details

Crystal data	
Chemical formula	(C_34_H_41_N_4_Se)_2_[Hg(CNSe)_4_]
*M*_r_	1789.84
Crystal system, space group	Monoclinic, *C*2/*c*
Temperature (K)	100
*a*, *b*, *c* (Å)	12.7788 (15), 27.276 (3), 20.180 (3)
β (°)	95.591 (2)
*V* (Å^3^)	7000.4 (15)
*Z*	4
Radiation type	Mo *K*α
μ (mm^−1^)	5.37
Crystal size (mm)	0.24 × 0.23 × 0.19

Data collection	
Diffractometer	Bruker APEXII CCD
Absorption correction	Multi-scan (*SADABS*; Bruker, 2002 ▸)
*T*_min_, *T*_max_	0.267, 0.336
No. of measured, independent and observed [*I* > 2σ(*I*)] reflections	46553, 9030, 8196
*R*_int_	0.095
(sin θ/λ)_max_ (Å^−1^)	0.676

Refinement	
*R*[*F* ^2^ > 2σ(*F* ^2^)], *wR*(*F* ^2^), *S*	0.040, 0.097, 1.03
No. of reflections	9030
No. of parameters	463
No. of restraints	147
H-atom treatment	H-atom parameters constrained
Δρ_max_, Δρ_min_ (e Å^−3^)	1.97, −2.10

Computer programs: *APEX2* (Bruker, 2005 ▸), *SAINT* and *XPREP* (Bruker, 2002 ▸), *SHELXT* (Sheldrick, 2015*a* ▸), *SHELXL2018* (Sheldrick, 2015*b* ▸) and *SHELXTL* (Sheldrick, 2008 ▸).

## supplementary crystallographic information

### Crystal data

**Table:**

(C_34_H_41_N_4_Se)_2_[Hg(CNSe)_4_]	*F*(000) = 3528
*M**_r_* = 1789.84	*D*_x_ = 1.698 Mg m^−^^3^
Monoclinic, *C*2/*c*	Mo *K*α radiation, λ = 0.71073 Å
*a* = 12.7788 (15) Å	Cell parameters from 9623 reflections
*b* = 27.276 (3) Å	θ = 2.4–28.8°
*c* = 20.180 (3) Å	µ = 5.37 mm^−^^1^
β = 95.591 (2)°	*T* = 100 K
*V* = 7000.4 (15) Å^3^	Prism, colorless
*Z* = 4	0.24 × 0.23 × 0.19 mm

### Data collection

**Table:**

Bruker APEXII CCD diffractometer	8196 reflections with *I* > 2σ(*I*)
ω scans	*R*_int_ = 0.095
Absorption correction: multi-scan (SADABS; Bruker, 2002)	θ_max_ = 28.7°, θ_min_ = 3.2°
*T*_min_ = 0.267, *T*_max_ = 0.336	*h* = −17→17
46553 measured reflections	*k* = −36→36
9030 independent reflections	*l* = −26→27

### Refinement

**Table:**

Refinement on *F*^2^	147 restraints
Least-squares matrix: full	Hydrogen site location: inferred from neighbouring sites
*R*[*F*^2^ > 2σ(*F*^2^)] = 0.040	H-atom parameters constrained
*wR*(*F*^2^) = 0.097	*w* = 1/[σ^2^(*F*_o_^2^) + (0.0353*P*)^2^ + 29.0826*P*] where *P* = (*F*_o_^2^ + 2*F*_c_^2^)/3
*S* = 1.03	(Δ/σ)_max_ = 0.002
9030 reflections	Δρ_max_ = 1.97 e Å^−^^3^
463 parameters	Δρ_min_ = −2.10 e Å^−^^3^

### Special details

**Table:**

Geometry. All esds (except the esd in the dihedral angle between two l.s. planes) are estimated using the full covariance matrix. The cell esds are taken into account individually in the estimation of esds in distances, angles and torsion angles; correlations between esds in cell parameters are only used when they are defined by crystal symmetry. An approximate (isotropic) treatment of cell esds is used for estimating esds involving l.s. planes.

### Fractional atomic coordinates and isotropic or equivalent isotropic displacement parameters (Å^2^)

**Table:**

	*x*	*y*	*z*	*U*_iso_*/*U*_eq_	Occ. (<1)
Hg1	0.500000	0.26306 (2)	0.750000	0.02668 (6)	
Se1	0.34755 (3)	0.22031 (2)	0.67740 (2)	0.03961 (11)	
Se2	0.60087 (4)	0.33443 (2)	0.68639 (3)	0.06039 (16)	
C1S	0.3559 (3)	0.15796 (14)	0.7105 (2)	0.0329 (8)	
N1S	0.3565 (3)	0.11791 (14)	0.7281 (2)	0.0530 (11)	
C2S	0.5511 (4)	0.32426 (16)	0.6025 (3)	0.0527 (14)	
N2S	0.5206 (5)	0.3180 (2)	0.5468 (3)	0.0818 (18)	
Se3	0.35746 (3)	0.55143 (2)	0.44746 (2)	0.02529 (8)	
C1	0.3163 (3)	0.49229 (12)	0.48583 (17)	0.0246 (7)	
N1	0.4520 (2)	0.50194 (11)	0.40157 (15)	0.0268 (6)	
N2	0.2460 (2)	0.57747 (10)	0.50876 (15)	0.0266 (6)	
C2	0.3607 (3)	0.44802 (12)	0.46652 (17)	0.0253 (7)	
N3	0.1405 (2)	0.56201 (11)	0.58694 (16)	0.0266 (6)	
C3	0.3253 (3)	0.40392 (13)	0.49273 (18)	0.0274 (7)	
H3	0.355121	0.373771	0.480319	0.033*	
N4	0.5031 (2)	0.42500 (11)	0.39049 (15)	0.0258 (6)	
C4	0.2474 (3)	0.40351 (12)	0.53656 (18)	0.0268 (7)	
C5	0.2061 (3)	0.44847 (12)	0.55594 (18)	0.0264 (7)	
H5	0.153811	0.448618	0.586325	0.032*	
C6	0.2404 (3)	0.49287 (12)	0.53141 (17)	0.0244 (7)	
C7	0.2071 (3)	0.35552 (12)	0.56442 (19)	0.0289 (7)	
C8	0.2489 (4)	0.35045 (15)	0.6371 (2)	0.0415 (10)	
H8A	0.220563	0.320554	0.655474	0.062*	
H8B	0.227426	0.378964	0.661992	0.062*	
H8C	0.325862	0.348547	0.640679	0.062*	
C9	0.2425 (5)	0.31052 (15)	0.5271 (3)	0.0593 (15)	
H9A	0.319392	0.310115	0.528966	0.089*	
H9B	0.212572	0.311945	0.480534	0.089*	
H9C	0.218126	0.280689	0.547898	0.089*	
C10	0.0862 (4)	0.35594 (18)	0.5592 (4)	0.0672 (18)	
H10A	0.060786	0.324118	0.573535	0.101*	
H10B	0.058519	0.362129	0.513002	0.101*	
H10C	0.062382	0.381828	0.587900	0.101*	
C11	0.2057 (3)	0.54262 (12)	0.54487 (18)	0.0253 (7)	
C12	0.1378 (3)	0.61244 (13)	0.57613 (19)	0.0276 (7)	
C13	0.0821 (3)	0.64909 (14)	0.6057 (2)	0.0352 (8)	
H13	0.038815	0.642379	0.640317	0.042*	
C14	0.0942 (3)	0.69620 (15)	0.5809 (2)	0.0417 (10)	
H14	0.057845	0.722574	0.599213	0.050*	
C15	0.1576 (3)	0.70585 (14)	0.5305 (2)	0.0389 (9)	
H15	0.162170	0.738592	0.514960	0.047*	
C16	0.2146 (3)	0.66971 (13)	0.5018 (2)	0.0325 (8)	
H16	0.258323	0.676713	0.467486	0.039*	
C17	0.2036 (3)	0.62215 (12)	0.52651 (19)	0.0266 (7)	
C18	0.0780 (3)	0.53739 (13)	0.63476 (18)	0.0275 (7)	
H18A	0.078020	0.557671	0.675445	0.033*	
H18B	0.110525	0.505402	0.647584	0.033*	
C19	−0.0355 (3)	0.52925 (14)	0.60471 (18)	0.0317 (8)	
H19C	−0.069910	0.561405	0.595627	0.038*	
H19D	−0.035131	0.511632	0.561841	0.038*	
C20	−0.0988 (3)	0.49959 (14)	0.65154 (19)	0.0336 (8)	
H20C	−0.084659	0.512629	0.697324	0.040*	
H20D	−0.174743	0.503795	0.637724	0.040*	
C21	−0.0724 (3)	0.44565 (15)	0.6518 (2)	0.0376 (9)	
H21C	0.003550	0.441622	0.665720	0.045*	
H21D	−0.085951	0.432855	0.605869	0.045*	
C22	−0.1347 (3)	0.41508 (18)	0.6977 (2)	0.0482 (11)	
H22D	−0.125713	0.428832	0.742783	0.072*	
H22E	−0.108927	0.381218	0.698705	0.072*	
H22F	−0.209348	0.415560	0.681153	0.072*	
C11A	0.4382 (3)	0.45556 (13)	0.42009 (17)	0.0257 (7)	
C12A	0.5610 (3)	0.45354 (14)	0.35008 (18)	0.0276 (7)	
C13A	0.6369 (3)	0.44094 (15)	0.30797 (18)	0.0303 (8)	
H13A	0.658408	0.407932	0.302826	0.036*	
C14A	0.6792 (3)	0.47904 (16)	0.2740 (2)	0.0360 (9)	
H14A	0.731050	0.472026	0.244678	0.043*	
C15A	0.6474 (3)	0.52805 (16)	0.2819 (2)	0.0355 (9)	
H15A	0.678307	0.553194	0.257699	0.043*	
C16A	0.5729 (3)	0.54032 (15)	0.3238 (2)	0.0325 (8)	
H16A	0.552318	0.573426	0.329275	0.039*	
C17A	0.5288 (3)	0.50248 (13)	0.35761 (18)	0.0278 (7)	
C18A	0.5203 (10)	0.3728 (3)	0.3997 (3)	0.0244 (14)	0.852 (8)
H18C	0.510020	0.364134	0.446248	0.029*	0.852 (8)
H18D	0.594009	0.365117	0.392479	0.029*	0.852 (8)
C19A	0.4470 (8)	0.3412 (3)	0.3527 (5)	0.0277 (9)	0.852 (8)
H19A	0.450494	0.352300	0.306284	0.033*	0.852 (8)
H19B	0.373681	0.345283	0.363775	0.033*	0.852 (8)
C20A	0.4781 (4)	0.2871 (3)	0.3588 (4)	0.0309 (12)	0.852 (8)
H20A	0.478389	0.276876	0.405842	0.037*	0.852 (8)
H20B	0.424614	0.267235	0.332065	0.037*	0.852 (8)
C21A	0.5855 (4)	0.27671 (18)	0.3354 (3)	0.0380 (11)	0.852 (8)
H21A	0.640296	0.291481	0.367370	0.046*	0.852 (8)
H21B	0.589821	0.292579	0.291671	0.046*	0.852 (8)
C22A	0.6079 (5)	0.2223 (2)	0.3291 (3)	0.0549 (16)	0.852 (8)
H22A	0.604420	0.206332	0.372400	0.082*	0.852 (8)
H22B	0.678187	0.217741	0.314583	0.082*	0.852 (8)
H22C	0.555409	0.207595	0.296356	0.082*	0.852 (8)
C18B	0.517 (6)	0.3685 (19)	0.411 (3)	0.025 (3)	0.148 (8)
H18E	0.592583	0.359205	0.414384	0.031*	0.148 (8)
H18F	0.490086	0.362428	0.454562	0.031*	0.148 (8)
C19B	0.455 (5)	0.3390 (17)	0.356 (3)	0.028 (3)	0.148 (8)
H19E	0.379655	0.348402	0.354008	0.034*	0.148 (8)
H19F	0.480373	0.347037	0.312626	0.034*	0.148 (8)
C20B	0.4652 (19)	0.2838 (16)	0.368 (2)	0.031 (3)	0.148 (8)
H20E	0.431322	0.275516	0.409056	0.037*	0.148 (8)
H20F	0.426915	0.266244	0.330620	0.037*	0.148 (8)
C21B	0.5777 (19)	0.2659 (10)	0.3768 (12)	0.036 (3)	0.148 (8)
H21E	0.577496	0.230268	0.386154	0.043*	0.148 (8)
H21F	0.614890	0.282350	0.415953	0.043*	0.148 (8)
C22B	0.639 (2)	0.2747 (12)	0.3169 (13)	0.042 (5)	0.148 (8)
H22G	0.700518	0.253336	0.319720	0.064*	0.148 (8)
H22H	0.661217	0.309070	0.316499	0.064*	0.148 (8)
H22I	0.593579	0.267534	0.275995	0.064*	0.148 (8)

### Atomic displacement parameters (Å^2^)

**Table:**

	*U*^11^	*U*^22^	*U*^33^	*U*^12^	*U*^13^	*U*^23^
Hg1	0.02158 (9)	0.01884 (9)	0.03905 (12)	0.000	−0.00001 (7)	0.000
Se1	0.03104 (19)	0.02352 (18)	0.0598 (3)	−0.00375 (14)	−0.01827 (18)	0.00848 (17)
Se2	0.0556 (3)	0.0361 (2)	0.0907 (4)	−0.0220 (2)	0.0137 (3)	0.0094 (3)
C1S	0.0283 (17)	0.0288 (18)	0.040 (2)	−0.0052 (14)	−0.0040 (15)	0.0027 (16)
N1S	0.061 (3)	0.0317 (19)	0.061 (3)	−0.0115 (17)	−0.018 (2)	0.0078 (18)
C2S	0.053 (3)	0.031 (2)	0.080 (4)	0.0182 (19)	0.033 (3)	0.033 (2)
N2S	0.110 (4)	0.076 (3)	0.066 (3)	0.052 (3)	0.038 (3)	0.045 (3)
Se3	0.02258 (15)	0.02116 (16)	0.03000 (18)	−0.00393 (12)	−0.00818 (13)	0.00354 (13)
C1	0.0241 (15)	0.0218 (15)	0.0252 (16)	−0.0042 (12)	−0.0106 (13)	0.0045 (13)
N1	0.0242 (13)	0.0267 (14)	0.0279 (15)	−0.0031 (11)	−0.0055 (11)	0.0037 (12)
N2	0.0218 (13)	0.0233 (13)	0.0332 (16)	−0.0014 (11)	−0.0054 (11)	0.0018 (12)
C2	0.0263 (16)	0.0235 (16)	0.0239 (16)	−0.0023 (13)	−0.0086 (13)	0.0013 (13)
N3	0.0264 (14)	0.0214 (13)	0.0309 (15)	−0.0033 (11)	−0.0034 (11)	0.0008 (12)
C3	0.0312 (17)	0.0218 (15)	0.0278 (17)	−0.0018 (13)	−0.0048 (14)	−0.0008 (13)
N4	0.0255 (13)	0.0251 (14)	0.0250 (14)	−0.0026 (11)	−0.0060 (11)	−0.0002 (11)
C4	0.0305 (17)	0.0209 (15)	0.0270 (17)	−0.0064 (13)	−0.0068 (13)	0.0006 (13)
C5	0.0267 (16)	0.0242 (16)	0.0265 (17)	−0.0043 (13)	−0.0062 (13)	−0.0007 (13)
C6	0.0232 (15)	0.0226 (15)	0.0250 (16)	−0.0017 (12)	−0.0106 (12)	0.0028 (13)
C7	0.0363 (18)	0.0184 (15)	0.0319 (18)	−0.0026 (13)	0.0025 (14)	−0.0024 (14)
C8	0.062 (3)	0.0283 (19)	0.034 (2)	−0.0092 (18)	0.0025 (19)	0.0041 (16)
C9	0.108 (4)	0.0217 (19)	0.054 (3)	−0.021 (2)	0.037 (3)	−0.0152 (19)
C10	0.036 (2)	0.039 (2)	0.123 (5)	−0.015 (2)	−0.010 (3)	0.028 (3)
C11	0.0218 (15)	0.0236 (15)	0.0282 (17)	−0.0026 (12)	−0.0103 (13)	−0.0001 (13)
C12	0.0273 (16)	0.0226 (16)	0.0309 (18)	−0.0013 (13)	−0.0067 (13)	0.0013 (14)
C13	0.040 (2)	0.0285 (18)	0.038 (2)	−0.0022 (16)	0.0052 (16)	0.0005 (16)
C14	0.045 (2)	0.0262 (18)	0.055 (3)	0.0020 (17)	0.0071 (19)	−0.0025 (18)
C15	0.038 (2)	0.0216 (17)	0.057 (3)	−0.0031 (15)	0.0064 (19)	0.0032 (17)
C16	0.0283 (17)	0.0247 (17)	0.044 (2)	−0.0057 (14)	−0.0006 (15)	0.0043 (16)
C17	0.0242 (15)	0.0202 (15)	0.0336 (18)	−0.0017 (12)	−0.0062 (13)	−0.0014 (13)
C18	0.0272 (16)	0.0271 (17)	0.0264 (17)	−0.0041 (13)	−0.0066 (13)	0.0012 (14)
C19	0.0244 (16)	0.039 (2)	0.0294 (18)	−0.0047 (14)	−0.0076 (14)	0.0019 (16)
C20	0.0272 (17)	0.043 (2)	0.0294 (18)	−0.0078 (15)	−0.0048 (14)	−0.0012 (16)
C21	0.0316 (19)	0.043 (2)	0.037 (2)	−0.0084 (16)	−0.0023 (16)	0.0050 (17)
C22	0.039 (2)	0.054 (3)	0.051 (3)	−0.012 (2)	0.0008 (19)	0.013 (2)
C11A	0.0243 (15)	0.0275 (16)	0.0236 (16)	−0.0007 (13)	−0.0061 (12)	0.0011 (13)
C12A	0.0242 (15)	0.0329 (18)	0.0234 (16)	−0.0031 (13)	−0.0100 (13)	0.0001 (14)
C13A	0.0249 (16)	0.039 (2)	0.0251 (17)	0.0003 (14)	−0.0074 (13)	0.0012 (15)
C14A	0.0230 (16)	0.053 (2)	0.0300 (19)	−0.0047 (16)	−0.0060 (14)	0.0019 (17)
C15A	0.0292 (18)	0.044 (2)	0.0311 (19)	−0.0082 (16)	−0.0074 (15)	0.0083 (17)
C16A	0.0280 (17)	0.0344 (19)	0.0331 (19)	−0.0037 (15)	−0.0071 (14)	0.0056 (16)
C17A	0.0234 (15)	0.0304 (17)	0.0274 (17)	−0.0021 (13)	−0.0084 (13)	0.0027 (14)
C18A	0.026 (2)	0.024 (2)	0.022 (3)	0.0008 (18)	−0.005 (3)	0.004 (2)
C19A	0.026 (2)	0.028 (2)	0.028 (2)	0.0015 (16)	−0.0036 (17)	−0.0035 (18)
C20A	0.034 (2)	0.027 (2)	0.030 (3)	−0.0015 (18)	−0.0016 (18)	−0.0035 (19)
C21A	0.043 (3)	0.037 (2)	0.034 (2)	0.013 (2)	0.002 (2)	−0.001 (2)
C22A	0.069 (4)	0.049 (3)	0.047 (3)	0.024 (3)	0.004 (3)	−0.005 (3)
C18B	0.026 (6)	0.024 (6)	0.026 (6)	0.003 (6)	−0.001 (6)	0.000 (6)
C19B	0.028 (5)	0.028 (5)	0.027 (5)	0.002 (5)	−0.004 (5)	−0.002 (5)
C20B	0.033 (5)	0.030 (5)	0.029 (5)	0.005 (5)	0.000 (5)	−0.004 (5)
C21B	0.039 (5)	0.034 (5)	0.033 (5)	0.005 (5)	−0.002 (5)	−0.004 (5)
C22B	0.042 (10)	0.048 (10)	0.037 (10)	0.011 (9)	0.003 (9)	−0.008 (9)

### Geometric parameters (Å, º)

**Table:**

Hg1—Se1	2.5972 (4)	C18—C19	1.533 (4)
Hg1—Se1^i^	2.5972 (4)	C18—H18A	0.9900
Hg1—Se2	2.7242 (5)	C18—H18B	0.9900
Hg1—Se2^i^	2.7242 (5)	C19—C20	1.533 (5)
Se1—C1S	1.826 (4)	C19—H19C	0.9900
Se2—C2S	1.771 (7)	C19—H19D	0.9900
C1S—N1S	1.149 (5)	C20—C21	1.509 (5)
C2S—N2S	1.165 (8)	C20—H20C	0.9900
Se3—C1	1.886 (3)	C20—H20D	0.9900
Se3—N1	2.087 (3)	C21—C22	1.527 (5)
Se3—N2	2.099 (3)	C21—H21C	0.9900
C1—C6	1.400 (5)	C21—H21D	0.9900
C1—C2	1.405 (5)	C22—H22D	0.9800
N1—C11A	1.336 (4)	C22—H22E	0.9800
N1—C17A	1.386 (5)	C22—H22F	0.9800
N2—C11	1.332 (5)	C12A—C13A	1.393 (5)
N2—C17	1.395 (4)	C12A—C17A	1.410 (5)
C2—C3	1.406 (5)	C13A—C14A	1.384 (6)
C2—C11A	1.443 (5)	C13A—H13A	0.9500
N3—C11	1.353 (5)	C14A—C15A	1.410 (6)
N3—C12	1.393 (4)	C14A—H14A	0.9500
N3—C18	1.473 (5)	C15A—C16A	1.375 (6)
C3—C4	1.394 (5)	C15A—H15A	0.9500
C3—H3	0.9500	C16A—C17A	1.387 (5)
N4—C11A	1.355 (5)	C16A—H16A	0.9500
N4—C12A	1.392 (5)	C18A—C19A	1.530 (5)
N4—C18A	1.450 (9)	C18A—H18C	0.9900
N4—C18B	1.60 (5)	C18A—H18D	0.9900
C4—C5	1.406 (5)	C19A—C20A	1.530 (5)
C4—C7	1.534 (5)	C19A—H19A	0.9900
C5—C6	1.395 (5)	C19A—H19B	0.9900
C5—H5	0.9500	C20A—C21A	1.520 (6)
C6—C11	1.461 (5)	C20A—H20A	0.9900
C7—C8	1.517 (6)	C20A—H20B	0.9900
C7—C9	1.531 (5)	C21A—C22A	1.520 (6)
C7—C10	1.537 (6)	C21A—H21A	0.9900
C8—H8A	0.9800	C21A—H21B	0.9900
C8—H8B	0.9800	C22A—H22A	0.9800
C8—H8C	0.9800	C22A—H22B	0.9800
C9—H9A	0.9800	C22A—H22C	0.9800
C9—H9B	0.9800	C18B—C19B	1.531 (9)
C9—H9C	0.9800	C18B—H18E	0.9900
C10—H10A	0.9800	C18B—H18F	0.9900
C10—H10B	0.9800	C19B—C20B	1.529 (9)
C10—H10C	0.9800	C19B—H19E	0.9900
C12—C13	1.394 (5)	C19B—H19F	0.9900
C12—C17	1.394 (5)	C20B—C21B	1.512 (10)
C13—C14	1.393 (6)	C20B—H20E	0.9900
C13—H13	0.9500	C20B—H20F	0.9900
C14—C15	1.387 (6)	C21B—C22B	1.519 (10)
C14—H14	0.9500	C21B—H21E	0.9900
C15—C16	1.386 (6)	C21B—H21F	0.9900
C15—H15	0.9500	C22B—H22G	0.9800
C16—C17	1.402 (5)	C22B—H22H	0.9800
C16—H16	0.9500	C22B—H22I	0.9800
			
Se1—Hg1—Se1^i^	126.637 (19)	H19C—C19—H19D	108.0
Se1—Hg1—Se2	114.732 (19)	C21—C20—C19	112.7 (3)
Se1^i^—Hg1—Se2	102.907 (18)	C21—C20—H20C	109.1
Se1—Hg1—Se2^i^	102.908 (18)	C19—C20—H20C	109.1
Se1^i^—Hg1—Se2^i^	114.732 (19)	C21—C20—H20D	109.1
Se2—Hg1—Se2^i^	88.78 (3)	C19—C20—H20D	109.1
C1S—Se1—Hg1	101.43 (11)	H20C—C20—H20D	107.8
C2S—Se2—Hg1	101.31 (14)	C20—C21—C22	113.9 (4)
N1S—C1S—Se1	175.7 (4)	C20—C21—H21C	108.8
N2S—C2S—Se2	178.3 (5)	C22—C21—H21C	108.8
C1—Se3—N1	79.95 (14)	C20—C21—H21D	108.8
C1—Se3—N2	79.34 (14)	C22—C21—H21D	108.8
N1—Se3—N2	159.29 (11)	H21C—C21—H21D	107.7
C6—C1—C2	121.1 (3)	C21—C22—H22D	109.5
C6—C1—Se3	119.9 (3)	C21—C22—H22E	109.5
C2—C1—Se3	119.0 (3)	H22D—C22—H22E	109.5
C11A—N1—C17A	108.1 (3)	C21—C22—H22F	109.5
C11A—N1—Se3	113.0 (2)	H22D—C22—H22F	109.5
C17A—N1—Se3	138.9 (2)	H22E—C22—H22F	109.5
C11—N2—C17	107.6 (3)	N1—C11A—N4	110.9 (3)
C11—N2—Se3	113.7 (2)	N1—C11A—C2	115.8 (3)
C17—N2—Se3	138.6 (2)	N4—C11A—C2	133.4 (3)
C1—C2—C3	118.5 (3)	N4—C12A—C13A	131.4 (4)
C1—C2—C11A	112.3 (3)	N4—C12A—C17A	106.8 (3)
C3—C2—C11A	129.2 (3)	C13A—C12A—C17A	121.8 (4)
C11—N3—C12	107.2 (3)	C14A—C13A—C12A	116.5 (4)
C11—N3—C18	129.7 (3)	C14A—C13A—H13A	121.8
C12—N3—C18	123.1 (3)	C12A—C13A—H13A	121.8
C4—C3—C2	121.4 (3)	C13A—C14A—C15A	121.7 (4)
C4—C3—H3	119.3	C13A—C14A—H14A	119.2
C2—C3—H3	119.3	C15A—C14A—H14A	119.2
C11A—N4—C12A	107.3 (3)	C16A—C15A—C14A	121.6 (4)
C11A—N4—C18A	129.7 (5)	C16A—C15A—H15A	119.2
C12A—N4—C18A	122.8 (5)	C14A—C15A—H15A	119.2
C11A—N4—C18B	122 (3)	C15A—C16A—C17A	117.4 (4)
C12A—N4—C18B	129 (3)	C15A—C16A—H16A	121.3
C3—C4—C5	118.7 (3)	C17A—C16A—H16A	121.3
C3—C4—C7	121.7 (3)	N1—C17A—C16A	132.1 (4)
C5—C4—C7	119.6 (3)	N1—C17A—C12A	107.0 (3)
C6—C5—C4	121.3 (4)	C16A—C17A—C12A	121.0 (4)
C6—C5—H5	119.4	N4—C18A—C19A	113.4 (6)
C4—C5—H5	119.4	N4—C18A—H18C	108.9
C5—C6—C1	119.0 (3)	C19A—C18A—H18C	108.9
C5—C6—C11	129.0 (3)	N4—C18A—H18D	108.9
C1—C6—C11	112.0 (3)	C19A—C18A—H18D	108.9
C8—C7—C9	108.0 (4)	H18C—C18A—H18D	107.7
C8—C7—C4	109.4 (3)	C18A—C19A—C20A	110.8 (4)
C9—C7—C4	112.2 (3)	C18A—C19A—H19A	109.5
C8—C7—C10	108.9 (4)	C20A—C19A—H19A	109.5
C9—C7—C10	108.4 (4)	C18A—C19A—H19B	109.5
C4—C7—C10	109.9 (3)	C20A—C19A—H19B	109.5
C7—C8—H8A	109.5	H19A—C19A—H19B	108.1
C7—C8—H8B	109.5	C21A—C20A—C19A	113.0 (5)
H8A—C8—H8B	109.5	C21A—C20A—H20A	109.0
C7—C8—H8C	109.5	C19A—C20A—H20A	109.0
H8A—C8—H8C	109.5	C21A—C20A—H20B	109.0
H8B—C8—H8C	109.5	C19A—C20A—H20B	109.0
C7—C9—H9A	109.5	H20A—C20A—H20B	107.8
C7—C9—H9B	109.5	C20A—C21A—C22A	113.0 (5)
H9A—C9—H9B	109.5	C20A—C21A—H21A	109.0
C7—C9—H9C	109.5	C22A—C21A—H21A	109.0
H9A—C9—H9C	109.5	C20A—C21A—H21B	109.0
H9B—C9—H9C	109.5	C22A—C21A—H21B	109.0
C7—C10—H10A	109.5	H21A—C21A—H21B	107.8
C7—C10—H10B	109.5	C21A—C22A—H22A	109.5
H10A—C10—H10B	109.5	C21A—C22A—H22B	109.5
C7—C10—H10C	109.5	H22A—C22A—H22B	109.5
H10A—C10—H10C	109.5	C21A—C22A—H22C	109.5
H10B—C10—H10C	109.5	H22A—C22A—H22C	109.5
N2—C11—N3	111.0 (3)	H22B—C22A—H22C	109.5
N2—C11—C6	114.9 (3)	C19B—C18B—N4	106 (3)
N3—C11—C6	134.1 (3)	C19B—C18B—H18E	110.5
N3—C12—C13	130.3 (4)	N4—C18B—H18E	110.5
N3—C12—C17	107.0 (3)	C19B—C18B—H18F	110.5
C13—C12—C17	122.7 (3)	N4—C18B—H18F	110.5
C14—C13—C12	115.4 (4)	H18E—C18B—H18F	108.7
C14—C13—H13	122.3	C20B—C19B—C18B	111.6 (13)
C12—C13—H13	122.3	C20B—C19B—H19E	109.3
C15—C14—C13	122.1 (4)	C18B—C19B—H19E	109.3
C15—C14—H14	118.9	C20B—C19B—H19F	109.3
C13—C14—H14	118.9	C18B—C19B—H19F	109.3
C16—C15—C14	122.7 (4)	H19E—C19B—H19F	108.0
C16—C15—H15	118.6	C21B—C20B—C19B	113.8 (13)
C14—C15—H15	118.6	C21B—C20B—H20E	108.8
C15—C16—C17	115.7 (4)	C19B—C20B—H20E	108.8
C15—C16—H16	122.1	C21B—C20B—H20F	108.8
C17—C16—H16	122.1	C19B—C20B—H20F	108.8
C12—C17—N2	107.2 (3)	H20E—C20B—H20F	107.7
C12—C17—C16	121.3 (3)	C20B—C21B—C22B	114.6 (13)
N2—C17—C16	131.5 (4)	C20B—C21B—H21E	108.6
N3—C18—C19	111.1 (3)	C22B—C21B—H21E	108.6
N3—C18—H18A	109.4	C20B—C21B—H21F	108.6
C19—C18—H18A	109.4	C22B—C21B—H21F	108.6
N3—C18—H18B	109.4	H21E—C21B—H21F	107.6
C19—C18—H18B	109.4	C21B—C22B—H22G	109.5
H18A—C18—H18B	108.0	C21B—C22B—H22H	109.5
C20—C19—C18	111.6 (3)	H22G—C22B—H22H	109.5
C20—C19—H19C	109.3	C21B—C22B—H22I	109.5
C18—C19—H19C	109.3	H22G—C22B—H22I	109.5
C20—C19—H19D	109.3	H22H—C22B—H22I	109.5
C18—C19—H19D	109.3		
			
N1—Se3—C1—C6	−179.9 (3)	Se3—N2—C17—C16	8.2 (6)
N2—Se3—C1—C6	0.1 (2)	C15—C16—C17—C12	1.0 (5)
N1—Se3—C1—C2	−1.6 (2)	C15—C16—C17—N2	178.2 (4)
N2—Se3—C1—C2	178.3 (3)	C11—N3—C18—C19	−96.6 (4)
C6—C1—C2—C3	1.5 (5)	C12—N3—C18—C19	80.9 (4)
Se3—C1—C2—C3	−176.6 (2)	N3—C18—C19—C20	174.9 (3)
C6—C1—C2—C11A	−179.1 (3)	C18—C19—C20—C21	−76.2 (4)
Se3—C1—C2—C11A	2.7 (4)	C19—C20—C21—C22	−179.7 (3)
C1—C2—C3—C4	0.5 (5)	C17A—N1—C11A—N4	0.3 (4)
C11A—C2—C3—C4	−178.7 (3)	Se3—N1—C11A—N4	−178.3 (2)
C2—C3—C4—C5	−1.7 (5)	C17A—N1—C11A—C2	179.7 (3)
C2—C3—C4—C7	178.9 (3)	Se3—N1—C11A—C2	1.1 (4)
C3—C4—C5—C6	1.0 (5)	C12A—N4—C11A—N1	−0.7 (4)
C7—C4—C5—C6	−179.6 (3)	C18A—N4—C11A—N1	175.0 (5)
C4—C5—C6—C1	1.0 (5)	C18B—N4—C11A—N1	171 (3)
C4—C5—C6—C11	178.9 (3)	C12A—N4—C11A—C2	−179.9 (3)
C2—C1—C6—C5	−2.3 (5)	C18A—N4—C11A—C2	−4.3 (7)
Se3—C1—C6—C5	175.9 (2)	C18B—N4—C11A—C2	−9 (3)
C2—C1—C6—C11	179.5 (3)	C1—C2—C11A—N1	−2.3 (4)
Se3—C1—C6—C11	−2.3 (4)	C3—C2—C11A—N1	176.9 (3)
C3—C4—C7—C8	106.9 (4)	C1—C2—C11A—N4	176.9 (3)
C5—C4—C7—C8	−72.5 (4)	C3—C2—C11A—N4	−3.9 (6)
C3—C4—C7—C9	−13.0 (5)	C11A—N4—C12A—C13A	−178.7 (3)
C5—C4—C7—C9	167.7 (4)	C18A—N4—C12A—C13A	5.3 (6)
C3—C4—C7—C10	−133.6 (4)	C18B—N4—C12A—C13A	11 (3)
C5—C4—C7—C10	47.0 (5)	C11A—N4—C12A—C17A	0.8 (3)
C17—N2—C11—N3	−1.5 (4)	C18A—N4—C12A—C17A	−175.3 (5)
Se3—N2—C11—N3	175.4 (2)	C18B—N4—C12A—C17A	−170 (3)
C17—N2—C11—C6	178.7 (3)	N4—C12A—C13A—C14A	179.5 (3)
Se3—N2—C11—C6	−4.4 (3)	C17A—C12A—C13A—C14A	0.2 (5)
C12—N3—C11—N2	1.0 (4)	C12A—C13A—C14A—C15A	0.2 (5)
C18—N3—C11—N2	178.8 (3)	C13A—C14A—C15A—C16A	0.1 (5)
C12—N3—C11—C6	−179.3 (3)	C14A—C15A—C16A—C17A	−0.7 (5)
C18—N3—C11—C6	−1.5 (6)	C11A—N1—C17A—C16A	180.0 (4)
C5—C6—C11—N2	−173.6 (3)	Se3—N1—C17A—C16A	−2.0 (6)
C1—C6—C11—N2	4.4 (4)	C11A—N1—C17A—C12A	0.2 (4)
C5—C6—C11—N3	6.7 (6)	Se3—N1—C17A—C12A	178.2 (2)
C1—C6—C11—N3	−175.3 (3)	C15A—C16A—C17A—N1	−178.6 (3)
C11—N3—C12—C13	179.1 (4)	C15A—C16A—C17A—C12A	1.2 (5)
C18—N3—C12—C13	1.1 (6)	N4—C12A—C17A—N1	−0.6 (3)
C11—N3—C12—C17	−0.2 (4)	C13A—C12A—C17A—N1	178.9 (3)
C18—N3—C12—C17	−178.1 (3)	N4—C12A—C17A—C16A	179.6 (3)
N3—C12—C13—C14	−177.4 (4)	C13A—C12A—C17A—C16A	−0.9 (5)
C17—C12—C13—C14	1.7 (6)	C11A—N4—C18A—C19A	90.1 (8)
C12—C13—C14—C15	−0.2 (6)	C12A—N4—C18A—C19A	−94.9 (10)
C13—C14—C15—C16	−1.0 (7)	N4—C18A—C19A—C20A	172.9 (7)
C14—C15—C16—C17	0.6 (6)	C18A—C19A—C20A—C21A	−65.4 (11)
N3—C12—C17—N2	−0.7 (4)	C19A—C20A—C21A—C22A	−169.3 (5)
C13—C12—C17—N2	179.9 (3)	C11A—N4—C18B—C19B	104 (4)
N3—C12—C17—C16	177.1 (3)	C12A—N4—C18B—C19B	−87 (6)
C13—C12—C17—C16	−2.2 (5)	N4—C18B—C19B—C20B	178 (4)
C11—N2—C17—C12	1.3 (4)	C18B—C19B—C20B—C21B	−55 (7)
Se3—N2—C17—C12	−174.3 (2)	C19B—C20B—C21B—C22B	−60 (4)
C11—N2—C17—C16	−176.1 (4)		

Symmetry code: (i) −*x*+1, *y*, −*z*+3/2.

### Hydrogen-bond geometry (Å, º)

**Table:**

*D*—H···*A*	*D*—H	H···*A*	*D*···*A*	*D*—H···*A*
C18—H18*A*···N1*S*^ii^	0.99	2.62	3.568 (5)	160
C18*A*—H18*C*···N2*S*	0.99	2.38	3.324 (8)	159
C18*B*—H18*F*···N2*S*	0.99	2.22	3.06 (6)	142

Symmetry code: (ii) −*x*+1/2, *y*+1/2, −*z*+3/2.

## References

[bb1] Back, T. G. (1999). *Organoselenium Chemistry: A Practical Approach.* Oxford University Press.

[bb2] Bhuyan, B. J. & Mugesh, G. (2012). *Biological and Biochemical Aspects of Selenium Compounds* In *Organoselenium Chemistry: Synthesis and Reactions* edited by T. Wirth, p. 361. Weinheim: Wiley-VCH.

[bb3] Bondi, A. (1964). *J. Phys. Chem.* **68**, 441–451.

[bb4] Brodersen, K., Cygan, M. & Hummel, H.-U. (1984). *Z. Naturforschung, Teil B*, **39**, 582-5.

[bb5] Bruker (2002). *SMART*, *SAINT* and *SADABS*. Bruker AXS Inc., Madison, Wisconsin, USA.

[bb6] Bruker (2005). *APEX2*. Bruker AXS Inc., Madison, Wisconsin, USA.

[bb7] Chan, M. H.-Y., Wong, H.-L. & Yam, V. W.-W. (2016). *Inorg. Chem.* **55**, 5570–5577. 10.1021/acs.inorgchem.6b00619 27206022

[bb8] Chivers, T. & Laitinen, R. S. (2015). *Chem. Soc. Rev.* **44**, 1725–1739. 10.1039/c4cs00434e 25692398

[bb9] Dorazco-González, A. (2014). *Organometallics*, **33**, 868–875.

[bb10] Fujihara, H., Mima, H. & Furukawa, N. (1995). *J. Am. Chem. Soc.* **117**, 10153–10154.

[bb43] Fujihara, H., Mima, H. & Furukawa, H. (1995). *J. Am. Chem. Soc.* **117**, 10153–10154.

[bb11] Kremer, A., Aurisicchio, C., De Leo, F., Ventura, B., Wouters, J., Armaroli, N., Barbieri, A. & Bonifazi, D. (2015). *Chem. Eur. J.* **21**, 15377–15387. 10.1002/chem.201501260 26471446

[bb12] Kushch, N. D., Buravov, L. I., Pesotskii, S. I., Lyubovskii, R. B., Yagubskii, E. B., Kaplunov, M. G., Golubev, E. V., Narymbetov, B. Zh., Khasanov, S. S., Zorina, L. V., Rozenberg, L. P., Shibaeva, R. P., Kobayashi, A. & Kobayashi, H. (1998). *J. Mater. Chem.* **8**, 897–901.

[bb13] Li, S.-L., Fun, H.-K., Chantrapromma, S., Wu, J.-Y. & Tian, Y.-P. (2006*a*). *Acta Cryst.* E**62**, i47–i49.

[bb14] Li, S.-L., Wu, J.-Y., Tian, Y.-P., Ming, H., Wang, P., Jiang, M.-H. & Fun, H.-K. (2006*b*). *Eur. J. Inorg. Chem.* pp. 2900–2907.

[bb15] Manjare, S. T., Kim, Y. & Churchill, D. G. (2014). *Acc. Chem. Res.* **47**, 2985–2998. 10.1021/ar500187v 25248146

[bb16] Mugesh, G. & Singh, H. B. (2000). *Chem. Soc. Rev.* **29**, 347–357.

[bb17] Poleschner, H. & Seppelt, K. (2004). *Chem. Eur. J.* **10**, 6565–6574. 10.1002/chem.200400596 15540265

[bb18] Pop, A., Silvestru, A., Juárez-Pérez, E. J., Arca, M., Lippolis, V. & Silvestru, C. (2014). *Dalton Trans.* **43**, 2221–2233. 10.1039/c3dt52886c 24301075

[bb19] Prasad, P. R., Selvakumar, K., Singh, H. B. & Butcher, R. J. (2016). *J. Org. Chem.* **81**, 3214–3226. 10.1021/acs.joc.6b00173 27010114

[bb20] Rani, V., Singh, H. B. & Butcher, R. J. (2017*a*). *Acta Cryst.* E**73**, 341–344. 10.1107/S2056989017001888 PMC5347049 28316804

[bb21] Rani, V., Singh, H. B. & Butcher, R. J. (2017*b*). *IUCrData*, **2**, x171746.

[bb22] Rani, V., Singh, H. B. & Butcher, R. J. (2017*c*). *Organometallics*, **36**, 4741–4752.

[bb23] Rani, V., Singh, H. B. & Butcher, R. J. (2018*a*). *Acta Cryst.* E**74**, 390–393. 10.1107/S2056989018002645 PMC5947810 29765730

[bb24] Rani, V., Singh, H. B. & Butcher, R. J. (2018*b*). *J. Organomet. Chem.* **859**, 33–43.

[bb25] Selvakumar, K., Shah, P., Singh, H. B. & Butcher, R. J. (2011*a*). *Chem. Eur. J.* **17**, 12741–12755. 10.1002/chem.201100930 21956838

[bb26] Selvakumar, K., Singh, H. B. & Butcher, R. J. (2011*b*). *Tetrahedron Lett.* **52**, 6831–6834.

[bb27] Selvakumar, K., Singh, H. B., Goel, N. & Singh, U. P. (2011*c*). *Organometallics*, **30**, 3892–3896.

[bb28] Selvakumar, K., Singh, V. P., Shah, P. & Singh, H. B. (2011*d*). *Main Group Chemistry*, **10**, 141–152.

[bb29] Sheldrick, G. M. (2008). *Acta Cryst.* A**64**, 112–122. 10.1107/S0108767307043930 18156677

[bb30] Sheldrick, G. M. (2015*a*). *Acta Cryst.* A**71**, 3–8.

[bb31] Sheldrick, G. M. (2015*b*). *Acta Cryst.* C**71**, 3–8.

[bb32] Shibaeva, R. P., Khasanov, S. S., Rozenberg, L. P., Kushch, N. D., Yagubskii, E. B. & Canadell, E. (1997). *Kristallografiya*, **42**, 846–850.

[bb33] Shibaeva, R. P., Rozenberg, L. P., Kushch, N. D. & Yagubskii, E. B. (1994). *Kristallografiya*, **39**, 825–831.

[bb34] Singh, V. P., Singh, H. B. & Butcher, R. J. (2011). *Chem. Commun.* **47**, 7221–7223. 10.1039/c1cc12152a 21623441

[bb35] Singh, F. V. & Wirth, T. (2012). *Selenium Compounds as Ligands and Catalysts* In *Organoselenium Chemistry: Synthesis and Reactions* edited by T. Wirth, pp. 321–334. Weinheim: Wiley-VCH.

[bb36] Space, G. & Armeanu, V. (1930). *Bul. Soc. Stiinte Cluj*, **5**, 294–318.

[bb37] Sun, H.-Q., Wang, X.-Q. & Zhang, W.-W. (2013). *Acta Cryst.* E**69**, i59. 10.1107/S1600536813022502 PMC3884434 24426978

[bb38] Sun, H.-Q., Yu, W.-T., Yuan, D.-R., Wang, X.-Q. & Xue, G. (2005). *Acta Cryst.* E**61**, i111–i112.

[bb39] Wang, Z., Sun, Z., Hao, X.-Q., Niu, J.-L., Wei, D., Tu, T., Gong, J.-F. & Song, M.-P. (2014). *Organometallics*, **33**, 1563–1573.

[bb40] Zade, S. S., Panda, S., Tripathi, S. K., Singh, H. B. & Wolmershäuser, G. (2004*a*). *Eur. J. Org. Chem.* pp. 3857–3864.

[bb41] Zade, S. S., Singh, H. B. & Butcher, R. J. (2004*b*). *Angew. Chem. Int. Ed.* **43**, 4513–4515. 10.1002/anie.200460380 15340957

[bb42] Zhao, L., Li, J., Li, Y., Liu, J., Wirth, T. & Li, Z. (2012). *Bioorg. Med. Chem.* **20**, 2558–2563. 10.1016/j.bmc.2012.02.049 22436386

